# Genomic Analysis of Third Generation Cephalosporin Resistant *Escherichia coli* from Dairy Cow Manure

**DOI:** 10.3390/vetsci4040057

**Published:** 2017-11-17

**Authors:** Muhammad Attiq Rehman, Xianhua Yin, Dion Lepp, Chad Laing, Kim Ziebell, Guylaine Talbot, Edward Topp, Moussa Sory Diarra

**Affiliations:** 1 Guelph Research and Development Center, Agriculture and Agri-Food Canada (AAFC), Guelph, ON N1G 5C9, Canada; attiq.muhammad@canada.ca (M.A.R.); Xianhua.Yin@AGR.GC.CA (X.Y.); Dion.Lepp@AGR.GC.CA (D.L.); 2 National Microbiology Laboratory at Lethbridge, Public Health Agency of Canada, Lethbridge, AB T1J 3Z4, Canada; chadr.laing@canada.ca; 3 National Microbiology Laboratory at Guelph, Public Health Agency of Canada, Guelph, ON N1G 3W4, Canada; Kim.Ziebell@phac-aspc.gc.ca; 4 Sherbrooke Research and Development Center, Agriculture and Agri-Food Canada (AAFC), Sherbrooke, QC J1M 0C8, Canada; Guylaine.Talbot@AGR.GC.CA; 5 London Research and Development Center, Agriculture and Agri-Food Canada (AAFC), London, ON N5V 4T3, Canada; Ed.Topp@AGR.GC.CA

**Keywords:** ceftiofur, cefotaxime, *E. coli*, dairy cow, manure, whole genome sequencing

## Abstract

The production of extended-spectrum β-lactamases (ESBLs) conferring resistance to new derivatives of β-lactams is a major public health threat if present in pathogenic Gram-negative bacteria. The objective of this study was to characterize ceftiofur (TIO)- or cefotaxime (FOX)-resistant *Escherichia coli* isolated from dairy cow manure. Twenty-four manure samples were collected from four farms and incubated under anaerobic conditions for 20 weeks at 4 °C or at 25 °C. A total of 37 TIO- or FOX-resistant *E. coli* were isolated from two of the four farms to determine their susceptibility to 14 antibiotics. Among the 37 resistant *E. coli*, 10 different serotypes were identified, with O8:H1 being the predominant serotype (*n* = 17). Five isolates belonged to each of serotypes O9:NM and O153:H42, respectively. All 37 cephalosporin resistant isolates were multi-resistant with the most prevalent resistance spectrum being amoxicillin-clavulanic acid-ampicillin-cefoxitin-ceftiofur-ceftriaxone-chloramphenicol-streptomycin-sulfisoxazole-tetracycline-trimethoprim-sulfamethoxazole. The genomes of 18 selected isolates were then sequenced and compared to 14 selected human pathogenic *E. coli* reference genomes obtained from public repositories using different bioinformatics approaches. As expected, all 18 sequenced isolates carried at least one β-lactamase *bla* gene: *TEM-1*, *TEM-81*, *CTX-M115*, *CTX-M15*, *OXA-1,* or *CMY-2*. Several other antibiotic resistance genes (ARGs) and virulence determinants were detected in the sequenced isolates and all of them harbored antimicrobial resistance plasmids belonging to classic Inc groups. Our results confirm the presence of diverse ESBL producing *E. coli* isolates in dairy cow manure stored for a short period of time. Such manure might constitute a reservoir of resistance and virulence genes for other bacteria that share the same environment.

## 1. Introduction

The dairy cow industry in Canada generates ~10 million kilograms of manure annually, which is applied to agricultural fields as fertilizer [1]. This manure is thus a valuable source of nutrients for crops; however, it can also be a source of manure-borne bacteria that could contaminate the environment and find their way into the food chain [2]. The bacterial pathogens that are commonly found in bovine manure can pose a major risk of infections for humans [3,4]. Dairy cows are frequently treated with cephalosporin antibiotics to prevent or cure mastitis that is caused largely by *Escherichia coli* or other pathogenic bacteria [5,6,7]. Thus, dairy cow manure could be an important reservoir of several antibiotic resistant bacteria (ARB) and their corresponding antibiotic resistance genes (ARGs) (collectively known as the resistome) [8]. A high prevalence of ARGs in commensal *E. coli* present in dairy cow manure reflects selection for resistance determinants and could have potential consequences on health if transferred to pathogenic bacteria [9]. Previous studies provided evidence that the majority of ARGs present in human pathogens have an environmental origin [10,11] and that ARB in manure were phylogenetically related to human pathogens [12]. Moreover, the land application of manure containing ARB could facilitate the dissemination of ARGs between bacteria via horizontal gene transfer [13]. Although extensive use of antibiotics increase the abundance of resistant bacteria in manure [9], it is also known that ARB could be abundant in manure from animals with no history of antibiotic use [2], indicating the natural presence of bacteria intrinsically resistant to antibiotics in the animal gut.

The knowledge of the origin, diversity, and transmission routes of ARGs will allow the implementation of mitigation strategies against the spread of resistant bacteria. In Gram-negative bacteria, resistance to β-lactam antibiotics is mainly mediated by the AmpC-like β-lactamases encoded by the *bla*_CMY-2_ gene and extended-spectrum β-lactamases (ESBLs), such as *bla*_TEM_, *bla*_SHV_, and *bla*_CTM_ genes [14,15]. The presence of these genes on mobile genetic elements (plasmids, transposons, and integrons) may be horizontally transferred to other bacteria [16,17]. ESBLs are produced to inactivate oxyimino cephalosporins (cefuroxime, cefotaxime, ceftazidine, ceftriaxone), and could be genetically linked to quinolones and aminoglycosides resistance (multi-drug resistance). Moreover, alteration in bacterial outer membrane protein porins by mutations can inhibit the diffusion of antibiotics across the bacterial membrane to a rate slow enough to facilitate the action of ESBL and AmpC-like enzymes [18]. The emergence of these *bla* genes in Gram negative bacteria found in dairy cows constitutes a new challenge [19,20,21]. The presence of *E. coli* resistant to extended-spectrum cephalosporins, such as ceftiofur in dairy production, can be an important food safety and public health issue. Several different approaches, including polymerase chain reaction (PCR), quantitative PCR (qPCR) of resistance genes, and analysis of plasmids, especially those carrying the β-lactamases, tetracycline, and sulfonamide resistance genes have been used previously to gain some insight into the manure resistome [22].

In the present study, we characterized dairy *E. coli* resistant to third generation cephalosporins (ceftiofur: TIO or cefotaxime: FOX) by whole genome sequencing (WGS) in order to gain insight into their resistance phenotype and genotype, as well as to their virulence potential. Detailed genomic analysis of such isolates and their comparison to pathogenic *E. coli* strains would help in understanding the ecology and epidemiology of this bacterium. We also determined how closely antibiotic resistance genotypes corresponded to the antibiotic resistance phenotypes.

## 2. Materials and Methods

### 2.1. Bacterial Isolation and Identification

A total of 24 samples, including six replicates/farm of liquid cow manure were collected from four dairy farms (C1 to C4) in the fall of 2013. At each location, the samples were collected at three randomly chosen locations within the stored manure at a depth of approximately one meter using sterilized Nalgene bottles attached to an adjustable sampling pole. Samples were incubated under anaerobic conditions for over 20 weeks at different temperatures (4 °C, or at 25 °C) to mimic seasonal variations. Sub-samples were collected and cultured every four weeks on MacConkey agar (Oxoid, Nepean, ON, Canada) supplemented with or without 4 μg/mL of ceftiofur (TIO) or cefotaxime (FOX), respectively, for an overnight culture at 35 °C to estimate total aerobic Gram-negative β-lactam resistant population. Sub-samples were also enriched in EC-broth containing the same antibiotics (TIO or FOX) for six hours and then spread on MacConkey Agar supplemented with TIO or FOX for an overnight culture at 35 °C. From each sample, three to five presumptive *E. coli* colonies were randomly selected and frozen at −80 °C in tryptic soy broth (Becton Dickinson, Mississauga, ON, Canada) containing 25% glycerol for characterization. The identity of all isolates was determined by API-20E (bioMérieux, St-Laurent, QC, Canada), PCR of the specific targeted regions and WGS on a subset of isolates. Somatic (O), and flagellar (H) antigens of all *E. coli* isolates were then identified by standard tube agglutination methods for the identification of O1 to O181 and H1 to H56, as previously described [23]. All *E. coli* were also screened by PCR for *eaeA*, *stx1*, *stx2*, *e-hlyA and fliCh7* genes to verify their VTEC pathotype [24].

### 2.2. Minimal Inhibitory Concentrations

Minimum inhibitory concentrations (MICs) were determined for all isolates using the Sensititre broth microdilution automated system (Thermo Scientific^TM^, Mississauga, ON, Canada), according to the Clinical Laboratory Standards Institute’s (CLSI) guidelines with *E. coli* ATCC 25922 as the quality control strain (5). The following 14 antibiotics were included in the test panel: amoxicillin-clavulanic acid, ampicillin, ceftiofur, ceftriaxone, cefoxitin, chloramphenicol, ciprofloxacin, gentamicin, azithromycin, nalidixic acid, streptomycin, sulfisoxazole, tetracycline, and trimethoprim-sulfamethoxazole. The antibiotic susceptibilities were interpreted according to the breakpoints of the CLSI’s and the Canadian Integrated Program for Antimicrobial Resistance Surveillance (CIPARS) (5) guidelines.

### 2.3. DNA Extraction

Genomic DNA from each of the 18 selected *E. coli* isolates was extracted using GeneElute Bacterial Genomic DNA kit (Sigma-Aldrich, Oakville, ON, Canada) for WGS. The extracted DNA was stored in 1X TE buffer (pH 8.0), quantified by Invitrogen Qubit^®^ 2.0 Fluorometer (Invitrogen, Carlsbad, CA, USA). The quality of DNA was visualized by 1% agarose gel electrophoresis and the DNA was then stored at −20 °C until further use.

### 2.4. Genome Sequencing, Assembly and Pan-Genome Analysis

Sequencing libraries were prepared from 1 ng of genomic DNA with the Nextera XT kit (Illumina, Inc., San Diego, CA, USA) according to the manufacturer’s instructions. The resulting libraries were quantitated by qPCR using primers p5 (5′-AATGATACGGCGACCACCGAGAT-3′) and p7 (5′-CAAGCAGAAGACGGCATACGA-3′) specific for the Nextera XT adapters and a standard curve prepared from the PhiX control library (Illumina). Samples were normalized to 4 nM with 10 mM Tris (pH 8) and an 8 pmol pooled library was sequenced with a MiSeq instrument (Illumina) using the 600 bp v3 kit (Illumina).

Initially, the FASTQ files for each of the 18 genomes of this study were assessed for quality. The adapter sequences were removed using trimgalore v0.4.1, which utilizes the cutadapt program [25] and genomes were assembled using Spades v3.7.1 [26]. The pan-genome distribution, as well as single-nucleotide differences among shared genomic regions for isolates, were determined using Panseq [27]. For the phylogenetic tree, the snp.fasta file was subsequently used as input to FastTreeMP, where a maximum likelihood tree using the “Generalized Time Reversible” model of nucleotide substitution [28] was constructed for the 18 *E. coli* genomes sequenced in this study as well as 14 reference genomes that were obtained from the National Center for Biotechnology Information (NCBI) database (accessed October 2016). Genomes from the NCBI included: two strains of serotype O15:H7 (accession #s NZ_JHNI00000000, NZ_CP008957), O104:H4 (accession #s CP003297, CP003289), O26:H11 (accession #s AP010953, JHNT01000000), and O111:NM (accession #s NZ_JHGU00000000, NZ_JFGU00000000), one strain of each serotype O26:NM (accession #NZ_JHFB00000000), O103:H2 (accession #NC_013353), O103:H25 (accession #NZ_AGSG00000000), O103:H11 (accession # NZ_JHFY00000000), O111:H8 (accession # NZ_JHGR00000000), O165:H25 (accession #NZ_JHMM00000000).

### 2.5. Virulence and Antimicrobial Resistance Genes

The comprehensive set of *E. coli* virulence factors from the SuperPhy platform were used to create separate fasta files containing genes in the following five categories: adherence, flagella, iron utilization, secretion systems, and toxins [29]. Genes in each of these five categories were screened against the studied genomes using Panseq and an image for the distribution of factors from each category was generated. The Resistance Gene Identifier (RGI) version 3.0.1 (https://card.mcmaster.ca/analyze/rgi) was used to identify AMR determinants in the 18 study genomes [30]. The genomes were then compared to fourteen selected human pathogenic *E. coli* reference genomes that were obtained from public repositories using the SuperPhy platform (http://lfz.corefacility.ca/superphy/) to build a comprehensive set of virulence factors and to identify Antimicrobial Resistance (AMR) determinants with a minimum of 97% amino acid sequence identify.

BLAST Ring Image Generator (BRIG, http://sourceforge.net/projects/brig/) was used to visualize whole genome sequence comparisons of coding sequence identity between O8:H1 and O25:H11 with STEC strains O157:H7 and O104:H4, respectively.

### 2.6. Plasmids

The PlasmidFinder database (https://cge.cbs.dtu.dk/services/PlasmidFinder/) from the Centre for Genomic Epidemiology was used to screen the 18 genome assemblies [31]. This was accomplished by constructing a BLAST nucleotide database of the genomes, and screening them using BLAST and the plasmid_database.fsa file from the PlasmidFinder database. The BLAST output file was used to parse the original genomes and separate the plasmid sequences from the genomic sequences. These putative plasmids were annotated using Prokka v1.12-beta (http://www.vicbioinformatics.com/software.prokka.shtml) [32].

### 2.7. Statistical Analyses

Bacterial enumerations were log transformed and analyzed using a repeated measurement analysis of SAS (SAS Inc., Cary, NC, USA) from week 0 to 20. The association test of Cochran-Mantel-Haenszel was used to determine the relationship between bacterial characteristics (resistance phenotype and genotype) and farms and collection times using the FREQ procedure in SAS. Associations between phenotype and genotype were determined using Pearson’s chi-square and Fisher’s exact tests [33]. A *p*-value of 0.05 was used to declare significance.

### 2.8. Genome Sequence Accession Numbers

The draft whole genome sequences of the 18 *E. coli* strains have been deposited in GenBank under the Bio-Project no PRJNA387025, and their accession numbers are: NHQJ00000000, NHQI00000000, NHQH00000000, NHQG00000000, NHQF00000000, NHQE00000000, NHQD00000000, NHQC00000000, NHQB00000000, NHQA00000000, NHPZ00000000, NHPY00000000, NHPX00000000, NHPW00000000, NHPV00000000, NHPU00000000, NHPT00000000, NHPS00000000.

## 3. Results

### 3.1. Cephalosporin Resistant Escherichia coli

**Figure 1:**
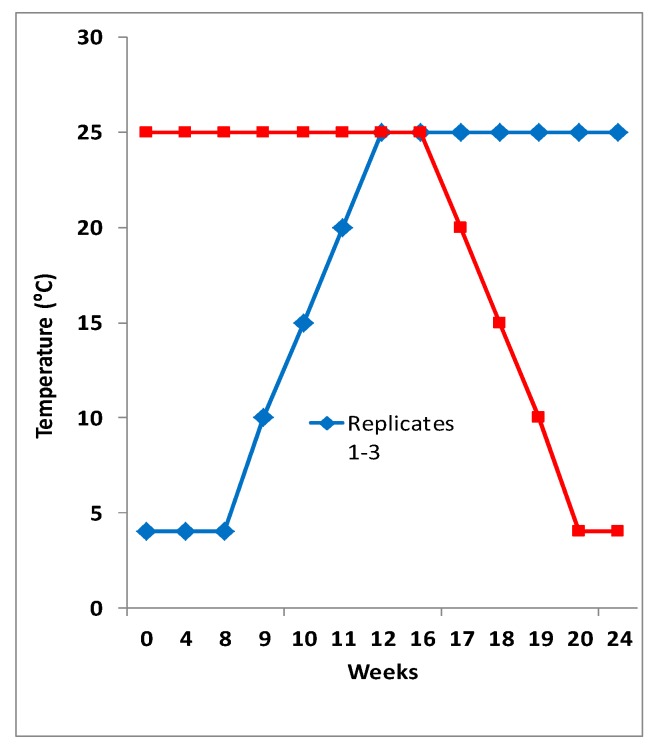
The temperature manipulations of manure samples. The graph shows isolation of ceftiofur or cefotaxime resistant Gram-negative bacteria from different manure samples incubated under anaerobic conditions for over 20 weeks at two temperature regimes (4 °C or at 25 °C). The gradual increase or decrease in temperature is shown in blue and red lines.

Manure samples from the four dairy farms were plated on MacConkey agar supplemented with TIO or FOX, and there was no significant difference between cephalosporin resistant Gram-negative bacterial counts. A total of 209 TIO- or FOX-resistant colonies were obtained. Of these, 37 (18%) were *E. coli*, and five were identified as *Acinetobacter baumannii* or *A. calcoaceticus* on the basis of API20E and PCR targeting *uidA* gene. Twenty-eight isolates were found to be *Pseudomonas oryzihabitans.* All 37 *E. coli* were isolated from manure samples of the farms C3 (*n* = 19) and C4 (*n* = 18) incubated at 4 °C during four (*n* = 11) or eight (*n* = 26) weeks. No TIO or FOX resistant *E. coli* was isolated from manures of farms C1 or C2 and from manure samples incubated during at least twelve weeks (Figure 1).

Traditional serotyping identified 10 different serotypes, with O8:H1 being the most prevalent serotype (*n* = 17) found exclusively in manure samples from C3. Six isolates of serotype O9:NM and five isolates of serotype O153:H42 were detected in manure samples from C4 (Table 1). None of the isolates were verotoxin-producing *E. coli* (VTEC, non-O157, negative for *eaeA*, *e-hlyA*, *fliC_h7_*, *stx1* or *stx2* gene) according to PCR results (data not shown) and based on whole genome sequence analysis on a subset of isolates. Our results showed the persistence of diverse cephalosporin resistant *E. coli* up to eight weeks of incubation at 4 °C in dairy cow manure.

### 3.2. Antibiotic Susceptibility

**Figure 2:**
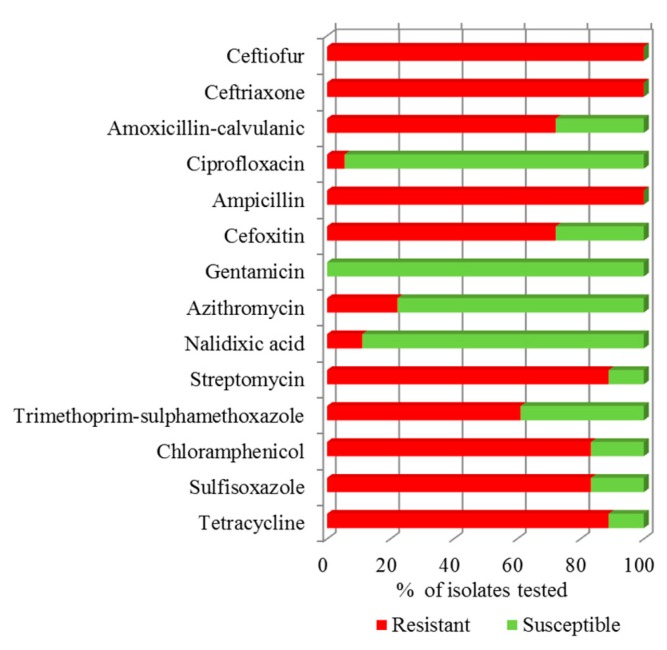
Antimicrobial Susceptibility test (AST). The AST results of the 18 *E. coli* selected isolates against 14 different antibiotics are presented as the percentage of isolates that were resistant (red), or susceptible (green) to the antibiotics listed on the *y*-axis.

All 37 TIO or FOX resistant *E. coli* isolates were also resistant to ceftriaxone and ampicillin. All but four were resistant to chloramphenicol and only two were susceptible to tetracycline. The history of antibiotic use by these farms is unknown. However, the prevalence for amoxicillin-clavulanic acid, cefoxitin, and trimethoprim-sulfamethoxazole resistance was significantly higher in isolates from C3 than those from C4 manure samples. Although none of these *E. coli* isolates were resistant to gentamicin, all were multi-drug resistant and the most prevalent resistance spectrum was amoxicillin-clavulanic acid-ampicillin-cefoxitin-ceftiofur-ceftriaxone-chloramphenicol-streptomycin-sulfisoxazole-tetracycline-trimethoprim-sulfamethoxazole. All five isolates of serotype O153:H42, the two isolates of serotype O25:H11 and the single isolate of serotype O33:H23 were susceptible to cefoxitin. An isolate of serotype O151:H9 was resistant to ciprofloxacin and nalidixic acid. Resistance to the latter antibiotic was also observed in one isolate of serotype O83:H42 (Figure 2 and Supplementary Table S1). These results show that TIO and FOX resistant *E. coli* from manure exhibited multiple-drug resistance phenotypes.

### 3.3. Sequencing, Assembly and Annotation

In order to assess the diversity of the virulence and antibiotic resistance determinants, genome of 18 representative isolates of the recovered 37 *E. coli* were sequenced, analyzed, and compared to those of 14 reference strains of *E. coli* from GenBank (National Center for Biotechnology Information, NCBI, accessed on October 2016). Genome size, number of predicted proteins, and putative plasmids for each of these 32 genomes are summarized in Table 2. Their Guanine-Cytosine (GC) content was around 51% for all of the genomes (data not shown), but their size and number of the predicted proteins varies, extensively. Illumina sequencing of the 18 isolates yielded from 0.26 to 1.08 billion nucleotides of DNA sequence, with an estimated average coverage ranging from 58× to 240×.

### 3.4. Virulence Factors

**Figure 3:**
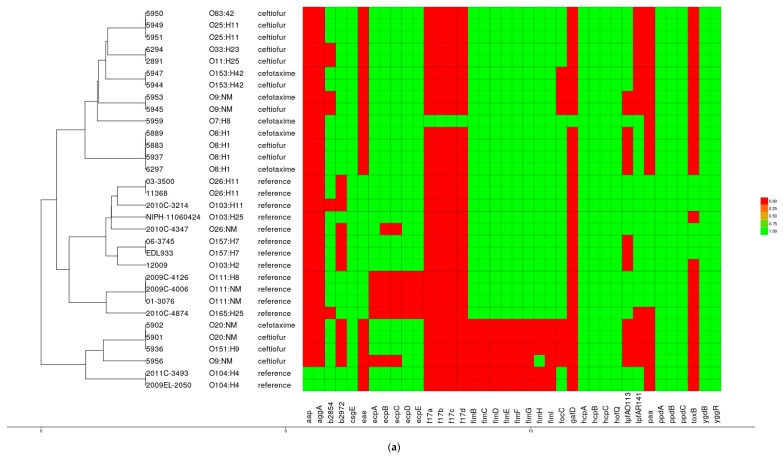

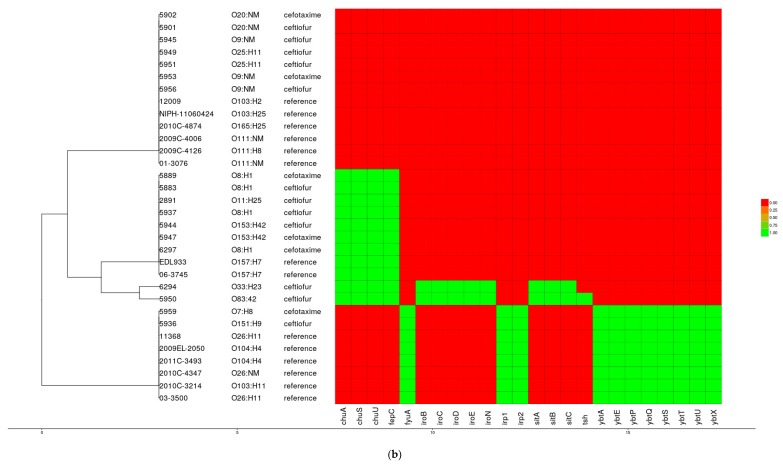

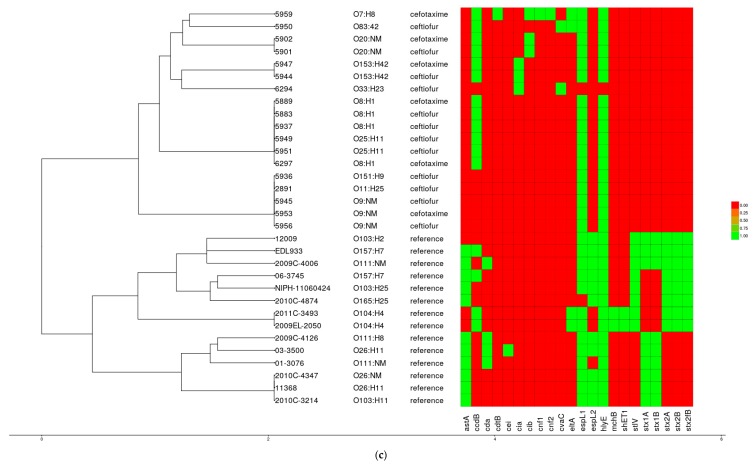

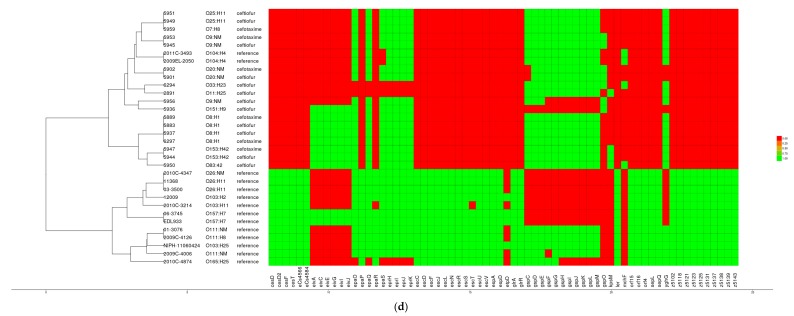
Heat map showing the presence (green color) and absence (red color) of (**a**) 38 adherence factors; (**b**) 24 iron uptake proteins; (**c**) 22 toxins; and, (**d**) secretions factors, across 18 TIO or FOX resistant from day cow manure and 14 pathogenic reference *E. coli* genomes. The bottom labels indicate the gene names. The tree on the left show a hierarchical clustering of the labelled strains based on the genes profile.

The 18 sequenced genomes were screened for virulence factors in the following categories: adherence, iron utilization, secretion systems, and toxins. Except for one isolate of serotype O7:H8, all of the others were negative for adhesion factors *f17a*, *f17b*, *f17c*, *f17d* (reported in *E. coli* from diarrheagenic and septicemic cows and human urinary tract infections cases) and *gafD.* This O7:H8 isolate harbored the most detected colonization factors (33 encoding genes). All 32 studied isolates (18 selected and 14 reference strains) clustered into three major groups based on the distribution of adherence factors. Four isolates (two of serotype O20:NM, and one each of serotype O151:H9 and O9:NM) shared more genes associated with adhesion also found in the pathogenic reference strain O104:H4. The *ecpA*/*B*/*C*/*D* cluster, a common pilus adherence factor for epithelial cell colonization was detected in all but isolate 5956 of serotype O9:NM. However, all 18 isolates were negative for *eae*, encoding the attaching and effacing protein intimin (Figure 3a), as also confirmed by PCR.

All 32 analyzed strains clustered into four distinct groups based on the distribution of 24 iron transport and utilization genes. Seven of the 18 studied isolates (four serotype O8:H1, two serotype O153:H42, and one serotype O11:H25) harbored the iron uptake genes (*chu*/*A*/*S*/*U*), and the ferric enterobactin transport ATP-binding protein (*fepC*). These isolates clustered with the reference O157:H7 strains. In addition to these four genes, isolates of both serotype O33:H23 and O83:H42 also harbored *iroCDEN*. Only two (serotype O7:H8 and O151:H9) of the studied isolates were positive for the ferric yersiniabactin uptake receptor *fyuA* (Figure 3b).

Two distinct clusters for 32 *E. coli* strains were observed based on the presence or absence of toxin genes; all 18 studied *E. coli* isolates clustered into a clade distinct from the 14 selected reference strains. However, a heat-labile enterotoxin-A subunit, which activates intracellular adenyl cyclase, was found in one of the reference strains of serotype O104:H4, as well as two studied isolates of serotype O7:H8 and a serotype O83:42. Additionally, the two isolates of serotype O7:H8, positive for eight toxin genes, contained a gene encoding cytotoxic necrotizing factors 1 and 2. Interestingly, all 32 strains were found to be positive for hemolysin E (*hlyE*), a novel pore-forming toxin and *espl1*, a putative type III secreted effector, with the exception of only one isolate of serotype O33:H23. Additionally, an operon *ccdB* that encodes Type II Toxin-antitoxin system was detected in a subset of the strains (Figure 3c).

All of the analyzed strains clustered into two major groups based on the genes involved in type III secretion systems (T3SS) and its associated regulatory genes. A widely conserved complete operon, *gspC*-*0*, which is similar in function to genes coding for components of the main terminal branch of the general secretory pathway and the genes required for type IV pilus biogenesis was detected in all but the isolates 5956 (O9:NM) and 5936 (O151:H9). Factors involved in secretory pathways were also detected in the majority of the strains (Figure 3d).

### 3.5. Antibiotic Resistance Genes

**Figure 4:**
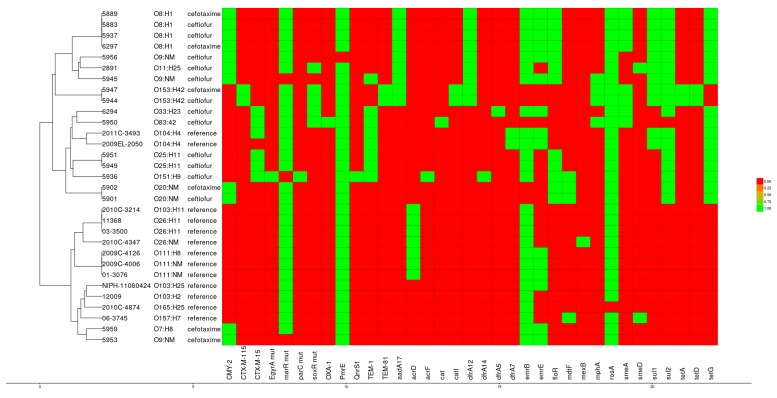
The heat map showing the presence (green color) and absence (red color) of antibiotic resistance genes (ARGs) across 18 TIO or FOX resistant and 14 pathogenic reference *E. coli* genomes. The bottom labels indicate the gene names. The tree on the left shows a hierarchical clustering of the labelled strains based on the genes profile.

The antibiotic resistance gene profiles of the selected 18 TIO or FOX resistant *E. coli* isolates identified by genomic analysis and reflecting their resistance phenotypes (Figure 2) are presented in Figure 4.

In agreement with their β-lactam-resistance phenotypes, all 18 selected isolates harbored at least one of the β-lactamase *bla* genes *TEM-1* (*n* = 6), *TEM-81* (*n* = 2), *CTX-M115* (*n* = 2), *CTX-M15* (*n* = 5), *OXA-1* (*n* = 1), and *CMY-2* (*n* = 11). Significant associations between the prevalence of these genes and serotypes were observed. For example, *CMY-2* was exclusively associated (*p* < 0.05) with serotypes O11:H25, O153:H42, O20:NM, O7:H8, O8:H1, and O9:NM, while *CTX-M15* were associated with serotype O151:H9, O25:H11, O33:H23, and O83:42 (*p* < 0.05). Several other ARGs, including multidrug resistance efflux pump systems, genes conferring resistance to cationic antimicrobial peptides, and polymyxin (*pmrE*), phenicols (*floR* and *cat*), aminoglycosides (*aadA17*, *strA*, *strB*), tetracyclines (*tetA*, *terD* and *tetG*), trimethoprim (*dfrA5*, *dfrA12* and *dfrA14*), sulfonamides (*sul1* and *sul2*), macrolides (*mphA*), and quinolones (*qnrs1*) were found in several isolates. In particular, an exceptionally high relative abundance of sulfonamide and tetracycline resistance genes, together with a set of genes that confer resistance to β-lactams and quinolones was observed. Of the 15 sulfonamide resistant *E. coli* isolates, *sul2* was detected in 83% of isolates. Similarly, *tetG* (78% of isolates) was the predominant tetracycline resistance genes found, while the remaining resistant isolates carried a combination of the *tetA* and *tetD* (Figure 4). Two isolates of serotype O153:H42 (IDs 5944 and 5947), and one of each serotype O9:NM (ID 5945) and O83:42 (ID 5950) harbored *mphA* conferring resistance to macrolides. Aminoglycoside resistance genes were detected in 15 of the 18 sequenced strains. Three isolates of each serotype O7:H8, O9:NM, and O83:42 (IDs: 5959, 5953, and 5950, respectively) showed susceptibility to aminoglycosides. Accordingly, no aminoglycosides resistance genes were detected in these isolates. Overall, the genomes of the 18 selected isolates contained a minimum of five ARGs. The detected ARGs in these 18 isolates correlated well with their observed resistance phenotypes (Table 3 and Table S1 in the supplemental material) as also confirmed by statistical analyses.

### 3.6. Mobile Genetic Elements (MGEs)

*Transposon*. A *Tn3*-like transposon, IS*1380*, widely distributed in *Acetobacter pasteurianus* and *Klebsiella pneumoniae*, was identified in several genomes of the 18 sequenced studied *E. coli* isolates. The transposons detected in eight of them showed a high sequence homology (>99%) to the transposon previously described in *Klebsiella pneumoniae* [34]. They harbored chromosomally anchored *bla*_CMY-2_ downstream of IS*1380* in six isolates and exhibited a high level of resistance against β-lactams. Other distinctive features of this transposon include the transposases (*TnpA*) and resolvase (*TnpR*) genes.

*Plasmid*. Several putative plasmids, including the *IncI*1 and *Inc*F type, of various sizes and composition were identified among the 18 sequenced genomes (Table 1). The *bla*_CMY-2_ carrying plasmids of various sizes were found in the two isolates of each serotype 20:NM (5901, 5902) and O9:MN (5945, 5953), as well as the serotype O7:H8 (5959). These five isolates were exclusively from farm C4.

**Figure 5:**
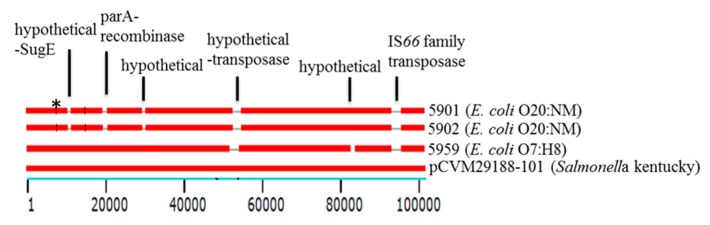
Nucleotide sequence alignments of *bla*_CMY-2_ containing plasmids identified in three sequenced *E. coli* isolates in this study to the previously characterized 101,461 bp plasmid pCVM29199-101 (GenBank accession # NC_011077) of *Salmonella* Kentucky isolated from poultry. The asterisk above the alignment indicates the position of class C β-lactamase *bla*_CMY-2_ present in all of the plasmids. The proteins absent in the plasmids are listed above the alignment. ParA and SugE are a chromosome partitioning and a quaternary ammonium compound-resistance protein, respectively. The *E. coli* isolates containing the plasmids are indicated on the right.

BLAST analysis of the *bla*_CMY-2_ containing contigs showed a significant similarity (97–100%) to previously described chromosomally integrated plasmids of *Salmonella* Heidelberg isolated from different environmental sources, including hospital settings in Canada (pSH146_65, GenBank accession # NC_019115.1, p12-4374_96, GenBank accession # NZ_CP012929.1). Interestingly, of the five isolates carrying the *bla*_CMY-2_ plasmids, the plasmid in three of them (5901 contig #20, 5902 # contig 30 and, 5959 contig #22) also exhibited 93% to 68% similarity to a well characterized virulence 101,461 bp chromosomally integrated plasmid from *Salmonella* Kentucky isolated from various poultry sources (pCVM29188_101, GenBank accession # NC_011077.1). In comparison to pCVM29188_101, all three *bla*_CMY-2_ harboring plasmids lack a region that encodes IS*66* family transposase. Two plasmids were also missing additional gene clusters encoding the chromosomal partitioning protein ParA, the quaternary ammonium compound-resistance protein SugE and several proteins of unknown functions (Figure 5).

The sequence type of the five *bla*_CMY2_ carrying plasmids was also determined by in silico plasmid multilocus sequence typing (pMLST) [31]. Two plasmids were identified as ST146 and ST12 types, and the other three were of unknown sequence type. Comparative analysis further revealed that one of the *bla*_CMY-2_ ST12-IncI1 plasmids from manure *E. coli* was 100% identical to those previously found in *Salmonella* from humans, but different from that of *E. coli* isolated from humans. The most frequently observed plasmid encoded ARGs were predicted to confer resistance to tetracycline, sulfonamides, streptomycin, and chloramphenicol. One plasmid from isolate 6297 (O8:H1) of the farm C3, and isolate 5953: (O9:NM) from the farm C4, encoded additional resistance to trimethoprim and bleomycin.

**Figure 6:**
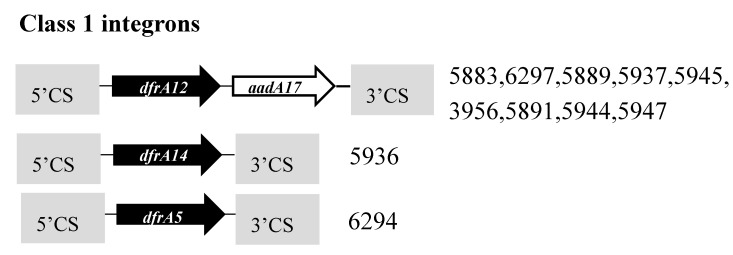
Schematic representation of the various gene cassette arrangements found in class 1 integrons detected in this study. The arrows display the open reading frames of the different genes. All *aadA* genes are presented as white arrows and *dfrA* genes as black arrows. The grey boxes indicate the 3′ and 5′ CSs regions typical of class 1 integrons harboring additional resistance determinants. On the right are the strains Ids containing the specific integron types. The gene cassettes are not drawn to scale.

*Integrons*: Ten (55%) of the 18 studied from farms C3 (six) and C4 (four) carried Class 1 (*intI1*) integron. Three types of gene cassette arrays were found among the Class 1 integron (Figure 6). The first type carried two gene cassettes, an *aadA17* coding for a 263 amino acid aminoglycoside adenyltransferase conferring resistance to streptomycin-spectinomycin and *dfrA12* coding for a 165 amino acid dihydrofolate reductase conferring resistance to trimethoprim. When compared with all other NCBI database entries, the deduced amino acid sequence of AadA17 was indistinguishable from those of several database entries, while DfrA12 showed two substitutions in comparison with the next closely related, same-sized DfrA12 proteins: Val-11 substituted for Ile and Glu-142 for Gln in the protein sequences of *E. coli*. The second type of Class 1 integron harbored a *dfrA14* coding for a 160 amino acid dihydrofolate reductase also conferring resistance to trimethoprim. The DfrA14 protein sequence was indistinguishable from those of several database entries. The third type of class 1 integron carried a *dfrA5* that encodes a 157 amino acid dihydrofolate reductase that is commonly found in *Vibrio cholera* [35] was detected in one isolate. The integron cassettes in the majority of isolates were also found associated with quaternary ammonium compound efflux SMR transporter *qacEΔ1* and sulfonamide resistance dihydropteroate synthase *sul1* genes.

### 3.7. Phylogenetic Analysis

**Figure 7:**
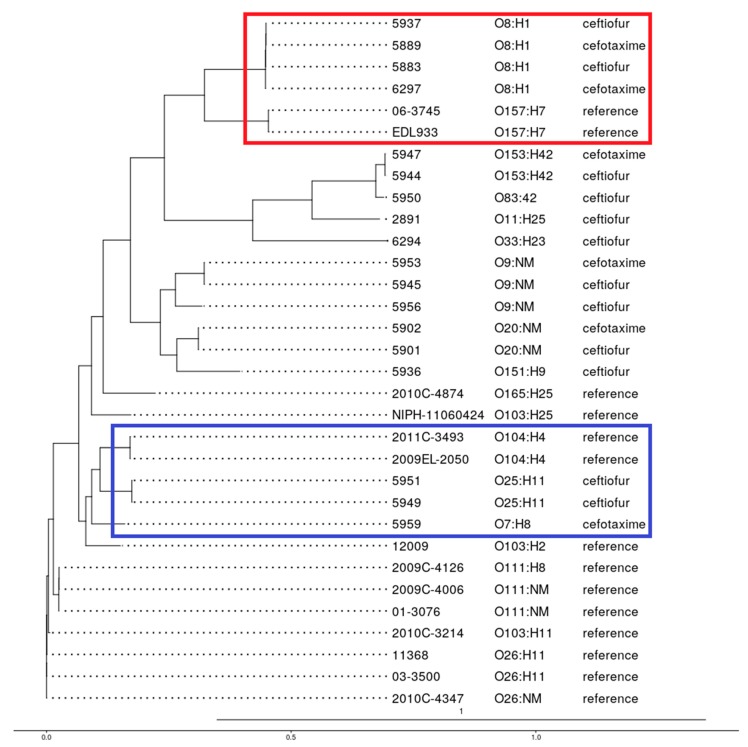
Genome-wide phylogenetic tree produced in PhyML using the “Generalized Time Reversible” model of nucleotide substitution showing the relationship among 18 *E. coli* isolates (of 10 different serotypes) recovered from dairy cow manure and their comparison with 14 reference genomes. The red and blue box show related strains. The scale is number of substitutions per site.

The phylogenetic analysis grouped all 18 TIO or FOX resistant isolates and the 14 selected reference strains into serotype specific clusters into six major clades. However, most of the reference strains grouped into two distinct clades. Four isolates of serotype O8:H1 clustered closely with the reference O157:H7 strains, while two isolates of serotype O25:H11 and one isolate of serotype O7:H8 grouped closely with two reference strains of serotype O104:H4. All of the other serotypes were found in discrete clades. Taken together, the single nucleotide variants (SNVs) based clustering revealed a common lineage among the non-STEC serotypes O8:H1, O7:H8, and O25:H11 with STEC strains O157:H7 and O104:H4, respectively (Figure 7).

### 3.8. Comparative Genomics

**Figure 8:**
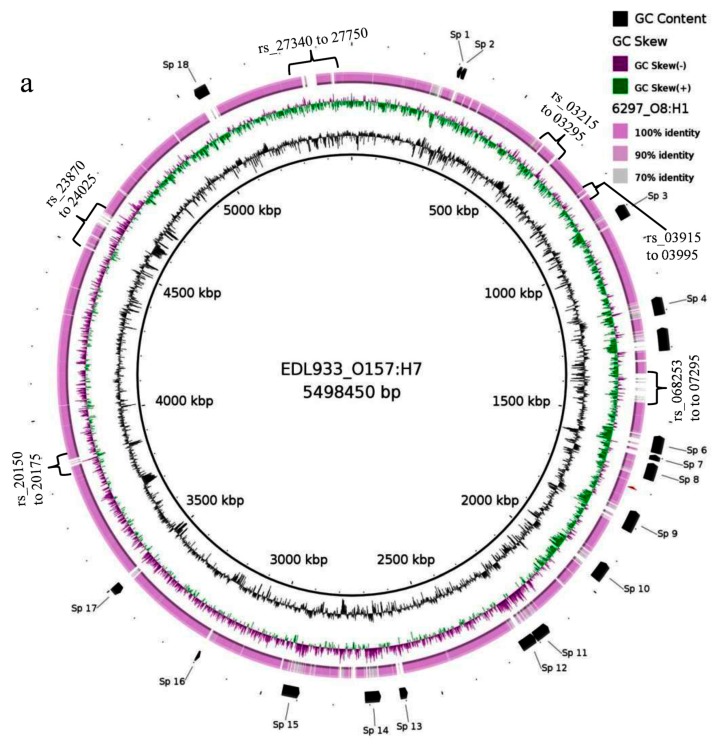

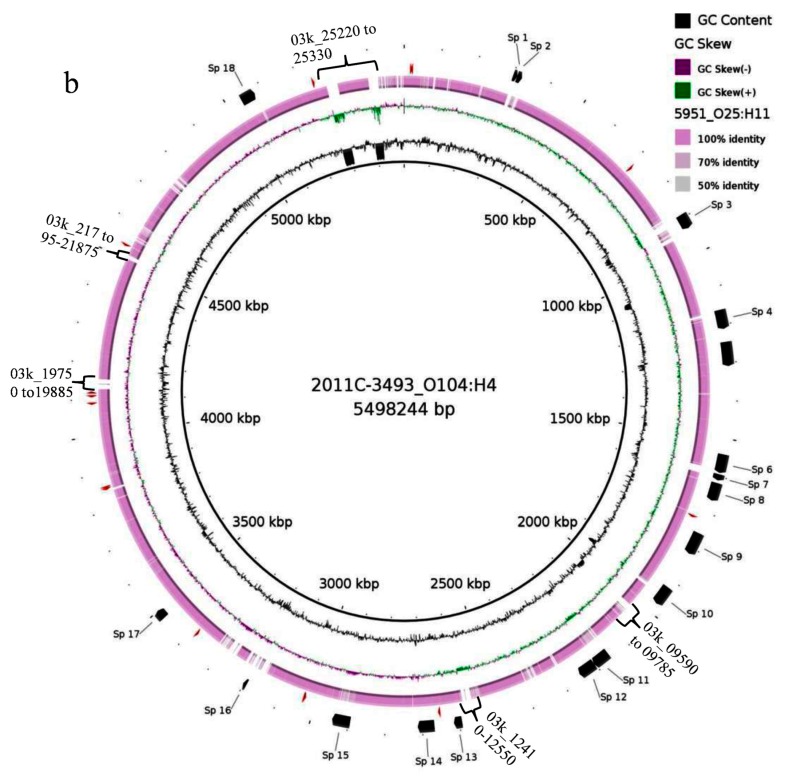
Comparative genomic analysis of (**a**) *E. coli* O157:H7 (strain EDL933) with *E. coli* O8:H1 (ID 6297) and (**b**) *E. coli* 0104:H4 (strain 2011C-3493) with *E. coli* O25:H11 (ID 5951). The innermost ring shows the reference genomes coverage. The innermost second and third rings show GC content and GC Skew. The outer ring (pink) shows the sequence homology with the reference genomes and the outermost ring highlights the SP sites (prophage regions) annotated in black. The circular plot was generated using BLAST Ring Image Generator (BRIG) software (http://brig.sourceforge.net/).

We compared the genomes of serotype O8:H1 (ID 6297, farm C3) and O25:H11 (ID 5951, farm C4) to the reference genomes of STEC producing strains O157:H7 (EDL933, GenBank accession # NZ_CP008957) and O104:H4 (2011C-3493, GenBank accession # CP003289) from the NCBI database using BRIG (http://brig.sourceforge.net/). The results of BRIG analyses, in which every open reading frame that is present in the reference genome, but absent in the genomes when compared, represented as a blank space are presented in Figure 8a,b. Even though small differences between the most similar strains were difficult to visualize, the isolate 6297 (O8:H1) clearly exhibited significant relatedness with the O157:H7 reference strain. Six O157:H7 genomic regions, ranging in size from 8.0 to 80.0 kb, were absent in the analyzed (labeled in Figure 8a, as rs_03215 to 03295, rs_03915 to 03995, rs_068253 to 07295, rs_20150 to 20175, rs_23870 to 24025, rs_27340 to 27750). Although these regions in O157:H7 primarily carry genes of unknown function, three were found to carry genes that were associated with cell envelope integrity, and one a type IV fimbrial gene.

Serotype O25:H11 showed the closest relationship with the O104:H4 reference strain, with the gene content of both of them being highly similar. However, five variable regions ranging in size from 7 to 50 Kb were identified (labeled in Figure 8b as 03k_09590 to 09785, 03k_12410-12550, 03k_19750 to19885, 03k_21795-21875, and 03k_25220-25330). Most of these regions carried genes that were hypothetical, involved in flagellar biosynthesis, transcriptional regulation, or Type 1 restriction modification systems.

The serotype O8:H1 and O:25:H11 shared several phage- and transposon-related loci with the respective reference strains. However, prophage carrying the Shiga-toxin 2 gene was not detected in the two cow manure isolates (O8:H1 and O:25:H11). These observations further support the segregation of O8:H1 and O:25:H11 s into distinct independent clades. Despite having highly similar gene content, variable regions were identified among the strains of this study.

## 4. Discussion

As reported in several previous studies, manure generated by dairy cattle may contain potentially harmful antibiotic resistant *E. coli* [36,37,38]. The emergence and spread of ESBL-producing *E. coli* associated with dairy cattle manure are of particular concern [39,40,41,42,43]. In the present study the antibiotic resistance profiles of TIO and FOX resistant *E. coli* isolates from dairy cattle manure samples were determined. Of the 209 TIO or FOX resistant isolates, 37 (18%) were identified as *E. coli*. They belonged to 10 non-VTEC serotypes with 76% of them belonging to three serotypes (O8:H1, O9:NM and O153:H42) . Since no TIO or FOX resistant *E. coli* were isolated from manure incubated longer than eight weeks at 4 °C to 25 °C or initially incubated at 25 °C, manure storage in an anaerobic condition for at least 12 weeks before handling and land spreading would be recommended. No *E. coli* on day 0 manure samples were identified, probably due to low level of this bacterium. Other identified bacteria included *Acinetobacter baumannii*, *Pseudomonas oryzihabitans*, *Ochrobactrum anthropic* and *Bordetella*/*Alcaligenes*/*Moraxella* spp. (data not shown).

All 37 *E. coli* isolates were sensitive to gentamicin, whereas 97% and 94.5% were sensitive to ciprofloxacin and nalidixic acid. Moreover, all were resistant to at least three classes of antibiotics. The serotype O151:H9 (isolate 5936) exhibited the worst AMR phenotype, showing resistance to 12 antibiotics while serotype O7:H8 (isolate 5959) showed the least AMR phenotype showing resistance to five of the 14 antibiotics tested. Among these multidrug resistant (MDR) isolates, 18 representative isolates were selected for whole genome sequence analysis to capture the range of resistance gene patterns and their phenotypic association, virulence determinants, as well as for genomic comparisons with pathogenic strains.

The sequence analysis allowed the detection of a large number of ARGs in the 18 selected *E. coli* isolates in agreement with other studies [8,44,45]. Reflecting their AMR phenotype, the isolate of serotype O153:H42 harbored a relatively large number of ARGs, while serotype O7:H8 carried the lowest number of resistance genes. In addition to multi-drug efflux pumps, five isolates contained *CTX-M15* and *TEM-1*, with one isolate also carrying *OXA-1*. As previously reported, *CTX-M15* has emerged recently in travel-related infections caused by pathogenic *E. coli* worldwide [46,47,48]. ESBL producing *Enterobacteriaceae* strains carrying *CTX-M* (ESBL-*CTX-M*) have been reported in healthy humans, and an ESBL-*CTX-M15 E. coli* B2:ST131 clone has been associated with hospital and community acquired infections [49]. The source of these bacteria needs to be identified in order to develop mitigation strategies. The contribution of food producing animals in the spread of ESBL-*CTX-M15 E. coli* to humans is not well established, however, the present study showed that multiple antibiotics resistant ESBL-*CTX-M15 E. coli* can be found in dairy cow manure.

Moreover, other ARGs, such as *bla*_CMY-2_, *floR* and or *dfrA1* on chromosome or on plasmids also were observed in several isolates. Three types of class 1 integron were detected in 10 of the 18 *E. coli* isolates. These integrons also harbor ARGs that can potentially be disseminated to other bacteria via horizontal gene transfer.

The chromosomal integrated *bla*_CMY-2_ containing plasmids identified in this study exhibited a high level of sequence similarity to plasmid pCVM29188-101 that is found in avian associated *Salmonella enterica* serotype Kentucky [50,51]. It could be speculated that this plasmid might be acquired from wild birds.

This study observed a significant association between the resistance phenotypes and genotypes among the 18 sequenced isolates. For individual antibiotics tested against each of the 18 isolates, there were 10 discrepancies observed and >96% agreements between the in silico AMR predictions and the wet-lab antimicrobial susceptibility testing. In agreement with other studies [52,53], these results suggest the robustness of WGS data in predicting the resistance phenotypes. The present study indicates that larger studies, including several farms, are warranted to investigate the prevalence of ESBL producing *Enterobacteriaceae* in dairy production environment.

Three serotypes, O8:H1, O153:H42, and O11:H25, harbored the virulence factors similar to O157:H7. Some of the detected virulence factors play an important role in biofilm formation during urinary tract infections, suggesting a virulence potential of the positive isolates. Recent studies have associated poultry isolates with urinary tract infections (UTIs) and that virulence may be determined by a complex of virulence factors [54,55]. Interestingly, small numbers of virulence factors observed in isolates carrying a large number of resistance genes. For example, serotype O7:H8 harbored the greater number of virulence genes but showed a lower resistance level and spectrum, which was confirmed by the resistance genotype. Moreover, the serotype O8:H1 showed a close phylogenetic relationship with O157:H7 and two other serotypes O7:H8 and O25:H11 were related to O104:H4 strain. These serotypes might have pathogenic potential and warrant further investigation.

The small number of genomes investigated (*n* = 18) constitutes a limitation of this study to determine accurate genotype to phenotype associations at large. However, the resistance genotype revealed by genome sequencing and analysis correlated well with the AMR phenotype of the studied isolates. It would be interesting to perform comparative analyses of isolates with contemporary epidemiologically related human clinical isolates to determine their true phylogenetic relationships. In addition, instead of long reads that can be generated by PacBio sequencing, the short reads from our Illumina MiSeq made the identification of plasmids and paralogues genes difficult.

## 5. Conclusions

The data from this study confirmed the presence of diverse *E. coli* serotypes harboring various ARGs, including ESBL*-CTX-M15* genes in dairy cow manures stored at 4 °C for up to eight weeks. The majority of the *E. coli* isolates were simultaneously resistant to β-lactams, quinolones, aminoglycosides, phenicols, trimethoprim, sulfonamides, and tetracyclines. Associations between genotypes and phenotypes were also observed and the detection of integrons and transposons indicated the potential of the dissemination of resistance genes. Genetic relationships were observed between non-STEC serotypes O8:H1 from manure and reference O157:H7 strains and between two serotypes (O25:H11 and O7:H8) and O104:H4 strains found in GenBank. Thus, stored dairy cow manure for a short period of time could constitute a reservoir of resistance genes and potential virulent *E. coli* that could be disseminated to other bacteria that are sharing the same environment. Further investigations are needed in large studies to determine the movement of ARGs from manure to the environment in the Canadian perspective, including the impact of manure treatments.

## Figures and Tables

**Table 1:** Serotyping of the 37 *E. coli* ^CFR^ isolates from manures of farm C3 and farm C4 incubated at 4 °C first.

Serotype	C3		C4		Total
	Week 4	Week 8	Week 4	Week 8	
O7:H8				1	1
O8:H1	8	9			17
O9:NM				6	6
O11:H25	1				1
O20:NM			2		2
O25:H11				2	2
O33:H23				1	1
O83:42				1	1
O151:H9		1			1
O153:H42				5	5

**Table 2:** Characteristics of the 32 isolates used in this study.

Strain ID	Serotype	Treatment	Genome Size (bp)	Coding Sequences	Source	Location	Year	Putative Plasmid
5883	O8:H1	ceftiofur	5,244,652	5230	Bovine manure	Canada	2014	IncA/IncFIB
5889	O8:H1	cefotaxime	5,238,639	5135	Bovine manure	Canada	2014	IncA/IncFIB
5891	O11:H25	ceftiofur	5,069,111	4957	Bovine manure	Canada	2014	IncA
5901	O20:NM	ceftiofur	4,776,338	4708	Bovine manure	Canada	2015	IncI1/IncQ1/IncFIA/IncFII
5902	O20:NM	cefotaxime	4,772,820	4698	Bovine manure	Canada	2015	IncI1/IncQ1/IncFIA/IncFII
5936	O151:H9	ceftiofur	4,869,431	4753	Bovine manure	Canada	2015	IncN/IncFIB
5937	O8:H1	ceftiofur	5,195,554	5061	Bovine manure	Canada	2015	IncA/IncFIB
5944	O153:H42	ceftiofur	5,101,758	4973	Bovine manure	Canada	2015	IncI1/IncQ1
5945	O9:NM	ceftiofur	4,766,659	4685	Bovine manure	Canada	2015	IncI2/IncFIB
5947	O153:H42	cefotaxime	5,101,804	4979	Bovine manure	Canada	2015	IncI1/IncQ1
5949	O25:H11	ceftiofur	4,994,147	5007	Bovine manure	Canada	2015	IncI1/IncQ1/IncFIA/IncFII
5950	O83:42	ceftiofur	5,311,653	5236	Bovine manure	Canada	2015	IncB/IncFIB/IncFIC/IncFII
5951	O25:H11	ceftiofur	4,992,789	4993	Bovine manure	Canada	2015	IncI1/IncQ1/IncFIA/IncFII
5953	O9:NM	cefotaxime	4,691,236	4600	Bovine manure	Canada	2015	IncI2
5956	O9:NM	ceftiofur	4,674,446	4565	Bovine manure	Canada	2015	IncI2/IncFII
5959	O7:H8	cefotaxime	5,198,736	5217	Bovine manure	Canada	2015	IncI1/IncFIB/IncFII
6294	O33:H23	ceftiofur	5,047,447	4959	Bovine manure	Canada	2015	IncI1/IncFIB/IncFII/IncQ1
6297	O8:H1	cefotaxime	5,188,526	5050	Bovine manure	Canada	2015	IncA/IncFIB
12009	O103:H2	reference	5,449,314	5121	Human stool	Japan	2001	IncFIB
11368	O26:H11	reference	5,697,240	5519	Human stool	Japan	2001	IncB/IncFIB/IncFII
NIPH-11060424	O103:H25	reference	5,159,902	5327	Human stool	Norway	2006	col156/IncFIB
2009EL-2050	O104:H4	reference	5,253,138	5183	Human stool	USA	2009	IncP/IncQ1/IncFIB/IncFII
2011C-3493	O104:H4	reference	5,273,097	5138	Human stool	USA	2011	IncP/IncQ/IncFIB/IncFII
2010C-4347	O26:NM	reference	5,293,499	5477	Not known	USA	2013	IncB/IncFIB
2010C-3214	O103:H11	reference	5,398,184	5493	Not known	USA	2013	IncB/IncFIB
06-3745	O157:H7	reference	5,316,785	5384	Human stool	USA	2013	IncFII
2010C-4874	O165:H25	reference	5,123,375	5211	Not known	USA	2013	IncFIB/IncFII
2009C-4006	O111:NM	reference	5,146,824	5263	Not known	USA	2013	col156/IncFII
03-3500	O26:H11	reference	5,337,704	5531	Not known	USA	2013	col156/IncFIB
2009C-4126	O111:H8	reference	5,093,584	5189	Not known	USA	2013	IncFII
01-3076	O111:NM	reference	5,038,636	5170	Not known	USA	2013	IncFII
EDL933	O157:H7	reference	5,547,323	5645	ground beef	USA	1982	IncFIB/IncFII

**Table 3:** Resistance phenotype and genotypes spectrum of selected 18 sequenced selected isolates used in this study.

Strain ID	Serotype	Resistance Phenotypes ^a^	Resistance Genotypes ^b^
5959	O7:H8	AMC, AMP, FOX, TIO, CRO	*bla*_CMY-2_
5883	O8:H1	AMC, AMP, FOX, TIO, CRO, STR, SXT, CHL, SUL, TET	*bla*_CMY-2_, *aadA-17*, *floR*, *dfrA-12*, *sul1-sul-2*, *tetG*, *strA*, *strB*
6297	O8:H1	AMC, AMP, FOX, TIO, CRO, STR, SXT, CHL, SUL, TET	*bla*_CMY-2_, *aadA-17*, *floR*, *dfrA-12*, *sul1-sul-2*, *tetG*, *strA*, *strB*
5889	O8:H1	AMC, AMP, FOX, TIO, CRO, STR, SXT, CHL, SUL, TET	*bla*_CMY-2_, *aadA-17*, *floR*, *dfrA-12*, *sul1-sul-2*, *tetG*, *strA*, *strB*
5937	O8:H1	AMC, AMP, FOX, TIO, CRO, STR, SXT, CHL, SUL, TET	*bla*_CMY-2_, *aadA-17*, *floR*, *dfrA-12*, *sul1-sul-2*, *tetG*, *strA*, *strB*
5945	O9:NM	AMC, AMP, FOX, TIO, CRO, AZM, STR, SXT, CHL, SUL, TET	*bla*_CMY-2_, *bla*_TEM1_, *aadA-17*, *floR*, *dfrA-12*, *sul1-sul-2*, *tetG*, *strA*, *strB*
5956	O9:NM	AMC, AMP, FOX, TIO, CRO, STR, SXT, CHL, SUL, TET	*bla*_CMY-2_, *aadA-17*, *floR*, *dfrA-12*, *sul1-sul-2*, *tetG*, *strA*, *strB*
5953	O9:NM	AMC, AMP, FOX, TIO, CRO	*bla*_CMY-2_
5891	O11:H25	AMC, AMP, FOX, TIO, CRO, STR, SXT, CHL, SUL, TET	*bla*_CMY-2_, *aadA-17*, *floR*, *dfrA-12*, *sul1-sul-2*, *tetG*, *strA*, *strB*
5901	O20:NM	AMC, AMP, FOX, TIO, CRO, STR, CHL, SUL, TET	*bla*_CMY-2_, *floR*, *sul1-sul-2*, *tetG*, *strA*, *strB*
5902	O20:NM	AMC, AMP, FOX, TIO, CRO, STR, CHL, SUL, TET	*bla*_CMY-2_, *floR*, *sul1-sul-2*, *tetG*, *strA*, *strB*
5949	O25:H11	AMP, TIO, CRO, STR, CHL, SUL, TET	*bla*_CTX-M15_, *bla*_TEM1_, *floR*, *dfrA-12*, *sul-2*, *tetG*, *strA*, *strB*
5951	O25:H11	AMP, TIO, CRO, STR, CHL, SUL, TET	*bla*_CTX-M15_, *bla*_TEM1_, *floR*, *dfrA-12*, *sul-2*, *tetG*, *strA*, *strB*
6294	O33:H23	AMP, TIO, CRO, STR, SXT, SUL, TET	*bla*_CTX-M15_, *bla*_TEM1_, *dfrA-5*, *sul-2*, *tetG*, *strA*, *strB*
5950	O83:42	AMC, AMP, TIO, CRO, AZM, NAL	*bla*_CTX-M15_, *bla*_TEM1_, *OXA-1*, *cat*, *tetG*
5936	O151:H9	AMC, AMP, CIP, TIO, CRO, NAL, STR, SXT, CHL, SUL, TET	*bla*_CTX-M15_, *bla*_TEM1_, *parC*, *qnrS1*, *floR*, *dfrA-14*, *sul-2*, *tetG*, *strA*, *strB*
5944	O153:H42	AMP, TIO, CRO, AZM, STR, SXT, CHL, SUL, TET	*bla*_CTX-M115_, bla*_TEM81_*, *aadA17*, *cat1*, *dfrA12*, *suI1-sul-2*, *tetA tetD*, *strA*,*strB*
5947	O153:H42	AMP, TIO, CRO, AZM, STR, SXT, CHL, SUL, TET	*bla*_CTX-M115_, bla*_TEM81_*, *aadA17*, *cat1*, *dfrA12*, *suI1-sul-2*, *tetA tetD*, *strA*, *strB*

**^a^** AMC, amoxicillin-clavulanic acid; AMP, ampicillin; AZM, azithromycin; FOX, cefoxitin; TIO, ceftiofur; CRO, ceftriaxone; STR, streptomycin; CHL, chloramphenicol; NAL, nalidixic acid; SUL, sulfisoxazole; SXT, trimethoprim-sulphamethoxazole; TET, tetracycline. Parentheses indicate intermediate resistance. **^b^** *aadA17*, streptomycin adenylytransferase; *bla*_CMY-2_, beta-lactamase; *bla*_CTX-M_, *bla*_TEM_, *OXA-1*, extended-spectrum β-lactamase (ESBL); *cat* & *floR*, chloramphenicol acetyltransferase; *strA-strB*, streptomycin phosphotransferase; *sul1-sul2*, dihydropteroate synthase; *tetA-tetD-tetG*, tetracycline efflux.

## Supplementary Materials

The following are available online at www.mdpi.com/2306-7381/4/4/57/s1, Table S1: Antibiotic susceptibility and resistance gene profiles.
